# On the Theory of Clonal Selection in Carcinogenic Transformation

**DOI:** 10.1038/bjc.1974.219

**Published:** 1974-11

**Authors:** P. M. Naha, M. Ashworth

## Abstract

**Images:**


*Br. J. Cancer* (1974) **30**, 448

### ON THE THEORY OF CLONAL SELECTION IN CARCINOGENIC TRANSFORMATION

P. M. NAHA AND M. ASHWORTH

*From the Paterson Laboratories, Christie Hospital and Holt Radium Institute, Manchester M20 9BX*

Received 2 July 1974. Accepted 9 August 1974

**Summary.—A temperature sensitive variant (ts13) of African green monkey kidney cell line, carrying a temperature sensitive lesion in thymidine metabolism, was transformed by 40 μg/ml of methylnitrosourea (MNU) at the restricted temperature of 39·5°C, whereas the same cell line was not transformed by MNU at the permissive temperature of 33°C. Results presented in this paper raise the possibility that clones of cells carrying a biochemical lesion might possibly contribute to carcinogenesis with respect to certain chemical carcinogens.**

UNLIKE clonal selection in cellular immunity (Burnet, 1959) where cells (lymphocytes) differentiated from the thymus carry out specialized functions, transformation in response to carcinogens is not known to be selective at the cellular level, though tissue or organ specificity of carcinogens *in vivo* has been known (Magee and Barnes, 1967; Druckrey *et al.*, 1967). Success in inducing *in vitro* transformation in mammalian cells by chemical carcinogens (Berwald and Sachs, 1963; Heidelberger and Iype, 1967; Sanders and Burford, 1967) has opened the possibility of studying the mode of action of these carcinogens at the cellular and molecular level (Heidelberger, 1964, 1970) and is expected to shed light on the question of clonal selection in a population of apparently homogeneous cells. Environmentally induced selective pressure on cells, as in the case of temperature sensitive conditional lethal mutants, might offer the opportunity to test the theory of clonal selection in carcinogenic transformation of mammalian cells. In this paper we describe the use of a temperature sensitive variant cell line that showed high frequency transformation induced by the carcinogen methylnitrosourea (MNU) at the restricted temperature, whereas the variant at the permissive temperature or the parental cell line showed no detectable change in cellular morphology under similar conditions.

In contrast to other chemical carcinogens, *e.g.* hydrocarbons (Berwald and Sachs, 1963; Heidelberger and Iype, 1967) and dimethylnitrosamine (Magee and Barnes, 1967; Sanders and Burford, 1967), methylnitrosourea requires " little enzymatic metabolism " before it can exert its carcinogenic effect (Magee and Barnes, 1967). It is also a powerful carcinogen *in vivo* (Druckrey *et al.*, 1967; Herrold, 1966; Leaver, Swann and Magee, 1969). Cells treated only briefly with MNU *in vitro* acquired a capacity for (morphologically) altered growth when cultured later, as well as an ability to grow as tumours in a heterologous host (Sanders and Burford, 1967). The induction of a " transformed " state *in vitro* could be attributed to at least 3 cellular mechanisms: (1) direct transformation resulting from interaction with some critical target in the cell, (2) selection of pre-existing malignant cells or (3) activation of an oncogenic virus by the chemicals. MNU is known to alkylate nucleic acids (Swann and Magee, 1968; Rosenkranz, Bitoon and Schmidt, 1968) and cause inhibition of protein synthesis (Kleihues and Magee, 1973); methylation of DNA-guanine base, both *in vivo* and *in vitro* (Lawley, 1966; Schoental, 1967), might account for its mutagenic property in mammalian cells where it has been reported to cause single gene mutations (Kao and Puck, 1971) but the continued lack of clear understanding of the relationship between mutagenesis and carcinogenesis testifies to the inadequacy of our knowledge on the one hand and systems employed for *in vitro* tests on the other. We therefore explored the possibility of using a system where the carcinogen could be used at sublethal doses on temperature sensitive cells in a conditionally defective state, and would thus allow an ideal control system where the same cells could be exposed to the carcinogen at a permissive temperature without the specific genetic defect. Temperature sensitive transformed clones arising " spontaneously " out of a normal population of MNU treated cultures of a mouse cell line have been reported (DiMayorea *et al.*, 1973) but, unlike that system, we have used a temperature sensitive cell line with known biochemical lesion.

### MATERIALS AND METHODS

*Cell lines.*—The cell lines used in these experiments are: the SV40 sensitive African green monkey kidney (epithelial) cell line of BSC-1 (Meyer *et al.*, 1962) and a temperature sensitive variant, ts13, isolated by the methods described before (Naha, 1973*c*). These cell lines grow as monolayers to confluence and are strongly contact-inhibited in culture. Culture conditions for these cell lines have been reported (Naha, 1973*c*). The variant clone ts13 has been found to undergo high frequency transformation by SV40 at the restricted temperature of 39·5°C (Naha, 1973*a*, *b*), but was lytic to the virus at the permissive temperature of 33°C. Preliminary experiments (Naha, 1973*b*) have indicated that the clone ts13 (spontaneous reversion frequency less than $10^{-4}$) was defective in the metabolism of exogenous thymidine and was resistant to $10^{-3}$ mol bromodeoxyuridine (BUDR) at 39·5°C; the clone was, however, sensitive to the same concentrations of BUDR at 33°C. These results (to be published elsewhere) suggested a temperature sensitive lesion in the thymidine kinase (TK) gene (Kit *et al.*, 1963; Littlefield, 1965) which is known to cause lethal incorporation of BUDR in DNA. Whether the enzyme thymidine kinase itself was temperature sensitive in the variant ts13 has not yet been determined. The clone ts13 was selected for this study from a number of other temperature sensitive variants which were not transformed by MNU under similar conditions.

*Chemicals.*—N - methyl - N - nitrosourea (MNU) and $^{14}$C-N-methyl-N-nitrosourea ($^{14}$C-MNU) were kindly supplied by Dr A. W. Craig. Samples of MNU were freshly prepared before use by dissolving in Hanks' BSS at pH 6·8. Before treatment with MNU, cells were washed with Hanks' BSS (pH 6·8), then exposed to required concentrations of MNU.

*Experimental procedures.*—Cells at a density of $3 \times 10^5$ in 6 ml volumes were plated in 25 cm$^2$ (30 ml) Falcon tissue culture flasks and incubated for 18 h (roughly one cell generation time) at 39·5°C. Cells were then washed in pre-warmed Hanks' BSS (pH 6·8) for 15 min. MNU at required concentrations in Hanks' BSS (pH 6·8) was added to the cultures and incubated for 2 h at 39·5°C, except in the case of the control which was kept in Hanks' BSS. At the end of the treatment MNU solutions were pipetted out and replaced with 5 ml of growth medium of L-15 (Naha, 1969, 1973*c*) for each flask and incubated at 33°C. Viability of cells was counted after 48 h. For this, trypsinized cells were centrifuged at 1500 rev/min and resuspended in 1 ml of growth medium and stained with trypan blue. Replicate culture flasks were incubated for 2–4 weeks at 33°C; foci of transformed cells were counted under the microscope (×54). The centre of a group of piled up cells was considered as a focus.

### RESULTS

### *Dose dependence in MNU transformation*

Viability counts of cells consequent upon exposure to MNU showed (Fig. 1)

FIG. 1.—Dose response to MNU in terms of total number of viable cells and transformation frequency in BSC-1 and the temperature sensitive variant ts13. Results from 2 separate experiments performed are presented. Viability counts of the parental cell line BSC-1 are represented in triangles, those of the variant ts13 in circles. The transformation frequency of ts13 is represented with bars to indicate variables, that of the parent cell line BSC-1 was nil (discontinuous line). Details in text.

that at concentrations below 80 μg/ml both cell lines were nearly 100% viable. The toxic (lethal) effect of the carcinogen began to appear at a concentration of 100 μg/ml; this is probably more true for the variant clone ts13 than in the case of the parental cell line BSC-1. At concentrations between 20 and 60 μg/ml of MNU, cells of ts13 appeared to be slightly stimulated in growth in repeated experiments which was not evident in the parental cell line. This stimulation in growth of ts13 was optimal at 40 μg/ml of MNU when cultures were transferred to 33°C after initial exposure to 39·5°C. The control cultures of ts13 incubated and treated at 33°C did not exhibit this stimulatory effect; in all respects these cultures behaved like the parental type.

Transformed cells (foci of uninhibited cells) began to appear in cultures of ts13, which were pre-incubated and treated with MNU at 39·5°C, between 1 and 2 weeks after MNU treatment (Fig. 2), though none appeared in the parental culture of BSC-1 even after longer periods of incubation. The frequency of transformation (Fig. 1) in cultures of ts13 was found to be optimal at a concentration of 60 μg/ml of MNU. It might be significant that the stimulating effect of the carcinogen in this cell line began to diminish at about this concentration. At 100 μg/ml of MNU only rare foci are visible and at 200 μg/ml no focus formation could be detected. Control cultures of ts13 incubated and treated at 33°C did not produce any transformed foci. Cloning of transformed cells from MNU treated cultures of ts13 (at 39·5°C) was found to be relatively easy. Lightly trypsinized cells in culture, when shaken vigorously, tended to release the clumped cells (foci) first, which were replated at suitable dilutions. These cultures grew as discrete colonies. Purification of the transformed clones was performed through 2 subsequent passages. Ten such transformed clones were purified from MNU treated cultures of ts13 (termed as

FIG. 2.—Growth characteristics of BSC-1 (Fig. A) and the temperature sensitive variant ts13 (Fig. B) cell lines after treatment with 40 µg/ml of MNU at 39·5°C and incubated for 14 days at 33°C in L-15 medium. Experimental procedures as in Fig. 1. ×43.

FIG. 3.—Early stages (7 days at 33°C) of clonal growth and morphology of an untransformed (Fig. A) and a transformed (Fig. B) cell line of ts13. ×43.

FIG. 4.—Dose response to MNU in terms of total number of viable cells and mitotic index in BSC-1 and the temperature sensitive variant ts13 under conditions of cellular selection at the restricted temperature. Cells at a density of $3 \times 10^5$ in 6 ml volumes were planted in 25 $cm^2$ (30 ml) Falcon tissue culture flasks and incubated for 18 h at 39·5°C. Cells were treated as in Fig. 1; only a set of duplicate cultures were maintained at 39·5°C after MNU treatment. Total number of viable cells were counted after 2, 4, and 6 days at the respective temperature; replicate cultures were stained simultaneously with Giemsa and cells in mitoses counted under microscope ($\times$ 15). Viability is represented by lines (Day 2, continuous lines; Day 4, dotted lines; Day 6, discontinuous lines), mitotic index by histograms (Day 2, black; Day 4, white; Day 6, black and white).

ts13/MNU1–10) for further studies. Figure 3 shows a visual comparison of the early stages of growth and clonal morphology of untransformed and transformed cells of ts13.

*Dose response and temperature controlled selection*

The above experiments on dose–response of MNU on BSC-1 and ts13 cell lines were repeated under conditions where replicate cultures were incubated at the restricted temperature of 39·5°C for equal lengths of time. This experiment was performed to determine the effect of temperature controlled selection on the viability and mitotic index of these cell lines after treatment with MNU. Cells were exposed to single treatments of MNU under identical conditions. One set of cultures of each cell line was transferred to the permissive temperature (33°C) and another set was retained at the restricted temperature (39·5°C). Viability and mitotic index were studied at 2, 4 and 6 days after MNU treatment. The results (Fig. 4) indicated that in the parental cell line of BSC-1 which was independent of any effect of temperature within this range (33°C and 39·5°C) there was no selective pressure on cells in cultures, and that both viability and mitotic index had remained constant for each concentration of MNU (Fig. 4A, B). In the case of the temperature sensitive clone ts13, however, the effect of restricted temperature (39·5°C) appeared to favour growth of cells at lower concentrations of MNU than those at higher concentrations. The stimulating effect of the carcinogen, as shown in the increased number of viable cells, shifted more towards cell cultures treated with 20 $\mu$g/ml of MNU on the 6th day from the original optimal effect at 40 $\mu$g/ml on the 2nd day at 39·5°C (Fig. 4D). This " selective " effect was also reflected on the mitotic indices in these cultures where nearly 50% increase in the mitotic index was observed on the 4th and 6th days after treatment with MNU at 20 $\mu$g/ml. Mitotic indices declined in all other cultures of ts13 during the progress of incubation at 39·5°C. These unexpected results appeared more intriguing considering the fact that the restricted temperature of 39·5°C was expected to have an equal effect on the cellular population at non-toxic levels of MNU. Though as yet we do not have any explanation for these results, it seems clear that a selection of some kind was working at the cellular level Whether this selection effect had any relevance to transformation was also not clear. It is, however, understandable that transformation could manifest itself only in the presence of cellular multiplication, and some stimulatory effect was therefore probably necessary. In replicate cultures of ts13 incubated at 33°C (Fig. 4C), this selection was not operating. In the light of available information on methylation of nucleic acids by MNU (Brookes and Lawley, 1961; Loveless and Hampton, 1969), it might be important to look into the extent of 0-6 alkylation of guanine under these conditions, which has been suspected to be related to carcinogenesis (Loveless, 1969), considering the fact that methylation of 7(N) position of guanine had been thought to be irrelevant to mutagenesis (Loveless, 1969).

*Kinetics of cellular selection*

These experiments were performed to determine whether transformation of cells of ts13 in culture was due to a selective effect or was non-selective in nature. For this purpose, the same cultures of BSC-1 and ts13 were exposed repeated (3 times) to 40 $\mu$g/ml of MNU every 24 h at 39·5°C and transferred to 33°C in normal growth medium as before. If the effect of MNU was selective, transformation frequency should remain constant. If, however, the effect of MNU was non-selective, transformation frequency should increase in ts13 with each additional exposure. The results (Fig. 5, 6) indicated that in the parental cell line of BSC-1 there was a gradual loss in the number of viable cells after each exposure to MNU. The viability counts in ts13, on the other hand, had remained more or less constant after the initial exposure to MNU; the number of transformed cells in this case also did not increase. We concluded from these results that transformation of ts13 cells at the restricted temperature was selective in nature and single exposure was enough to cause transformation among the selected population.

The relatively higher viability of cells of ts13, compared with BSC-1, after repeated exposure to MNU was difficult to interpret. In order to test if the cells of ts13 at 39·5°C had become resistant to further methylation by the same concentration of MNU (30 $\mu$g/ml), we repeated these experiments with $^{14}$C-MNU under identical conditions and looked for radioactivity counts in the acid-soluble (protein) and acid-insoluble (DNA and insoluble protein) fractions. We faced 2 limitations in performing this experiment: (1) we were restricted to using not more than $3 \times 10^5$ cells/ml to eliminate the background caused by leakiness

Fig. 5.—Response of repeated exposure to MNU (40 μg/ml) in terms of viability and tranformation frequency in BSC-1 and the temperature sensitive variant ts13. Cells at a density of 3 × $10^5$ in 6 ml volumes were planted in 25 $cm^2$ (30 ml) Falcon tissue culture flasks and incubated for 18 h at 39·5°C. Cells were treated similarly as in Fig. 1, except that the replicate cultures were put back at 39·5°C for 12 h before 2nd and 3rd exposures to MNU. This was performed to bring the cells to identical conditions at each exposure.

Viability was counted after 4 days of the initial treatment with MNU; foci of transformed cells counted under microscope (× 35) after 2–4 weeks of incubation at 33°C. Viability of the parental line BSC-1 is represented by light discontinuous line, that of the temperature sensitive variant ts13 by light continuous line; the transformation frequency is denoted by corresponding heavy lines.

of ts13 at 39·5°C (increase in leakiness due to increased cell density is a well known phenomenon), (2) we used 40 μg/ml of MNU which caused low incorporation of $^{14}$C-MNU in the cells. The results (Fig. 7), in spite of low radioactivity counts recovered, nevertheless did indicate that compared with the parental cell line of BSC-1 where repeated exposures to MNU caused increased incorporation of radioactivity both in DNA and protein, the variant cells of ts13 did not show any increase in incorporation of $^{14}$C-MNU either in the DNA or in the protein. As these results were obtained with 24 h intervals between treatments, an allowance should be made for excision loss of the label from the DNA and for the protein turnover. There is, of course, no reason to assume that this accounted for the difference between the 2 cell lines. It would appear from these results that the transformed cells of ts13 were resistant to further methylation at the same concentration and that it is only the drug resistant population in which—under such operational conditions—transformation can be observed.

### DISCUSSION

The results presented in this paper suggest that MNU induced transformation *in vitro* of variant clone ts13, carrying a genetical lesion in thymidine metabolism (possibly in the thymidine kinase gene), was probably directly related to its biochemical defect. A population of cells carrying identical lesions at the restricted temperature could only account for the high frequency of transformation in this cell line at the restricted temperature. Further experimentation on different cell lines would be required to confirm the

FIG. 6.—Pictures showing extent of growth after 7 days in Giemsa stained preparations of BSC-1 (Fig. A–D) and the temperature sensitive variant ts13 (Fig. E–H) after 3 exposures to MNU (40 $\mu$g/ml), corresponding to the experiment described in Fig. 5. The serial pictures represent the untreated controls and 1st, 2nd and 3rd MNU treatments.

FIG. 7.—Incorporation of radioactivity after repeated exposures to 40 μg/ml of $^{14}C$-MNU (1·2 mCi/mmol) in presence of equal concentrations of cold MNU. Cells at density of 3 × $10^5$ ml volumes were planted on Falcon petri dishes (60 × 15 mm) with 3 coverslips (10 × 35 mm) in each dish. Cells were treated similarly as in Fig. 1, except that the replicate cultures were put back at 39·5°C for 12 h before 2nd and 3rd exposures to MNU. This was performed to bring the cells to identical conditions at each exposure. After each treatment the cells were washed with cold Hanks' BSS, fixed in acetic acid : ethanol (1 : 3), washed with 5% cold trichloracetic acid (TCA) and 5% hot TCA. The coverslips were then washed in methanol and air dried. Radioactivity in TCA precipitable fraction (DNA + insoluble proteins) in the coverslips was counted in toluene based scintillation fluid; the TCA soluble fraction (protein) in toluene : Triton X (13 : 7).

The counts represent the total for 3 coverslips. Circles refer to DNA, triangles to protein. Open symbols represent BSC-1, closed symbols represent the variant ts13.

involvement of the thymidine kinase gene in MNU induced transformation; these experiments nevertheless show that temperature sensitive cells could ideally be used in the study of *in vitro* chemical carcinogenesis with respect to their mode of action. These results raise the possibility that only selected clones that vary genetically (spontaneous or induced) from the rest of the population of cells are liable to transformation, at least with respect to certain carcinogens. It is also possible that there are other contributing factors involved, *e.g.* progressive decay in certain controls of cells, of which we know very little. The crucial point in all studies on chemical carcinogenesis *in vitro* is the determination of the frequency of spontaneous mutants arising in a population of primary cultures; the methods for such a study are still lacking. Preliminary analysis of the MNU transformed clones of ts13 have indicated that, among other changes in their growth characteristics, all the clones (100%) have reverted to sensitivity to BUDR ($TK^+$), whereas the original clone ts13 from which they were derived was BUDR resistant ($TK^-$). Whether the genetical change (from $TK^-$ to $TK^+$) was an event concurrent with that of transformation by MNU, or was a separate event independent of the mechanism of transformation, has not yet been determined; revertants are now being studied for linkage between BUDR sensitivity and " flat " revertants.

This work was supported by grants from the Cancer Research Campaign and the Medical Research Council. We are very grateful to Dr A. W. Craig for stimulating discussion and indebted to him for the supply of MNU, and also thank Mrs. Kathleen Hewitt for excellent technical assistance.

### REFERENCES

BERWALD, Y. & SACHS, L. (1963) *In vitro* Cell Transformation with Chemical Carcinogens. *Nature, Lond.*, **200**, 1182.

BROOKES, P. D. & LAWLEY, P. (1961) The Alkylation of Guanosine and Guanylic Acid. *Biochem. J.*, **80**, 496.

BURNET, M. F. (1959) *The Clonal Selection Theory of Immunity*. Cambridge: Vanderbilt and Cambridge University Press.

DIMAYORCA, G., GREENBLATT, M., TRANTHER, T., SOLLER, A. & GIORDANO, R. (1973) Malignant Transformation of BHK21 Clone 13 Cells *in vitro* by Nitrosamines—a Conditional State. *Proc. natn. Acad. Sci. U.S.A.*, **70**, 46.

DRUCKREY, R., PREUSSMANN, R., IVANKOVIC, S. & SCHMÄHL, D. (1967) Organotrope carcinogene Wirkungen bei 65 verschidenen N-nitrosoverbindugen an BD-Ratten. *Z. Krebsforsch.*, **69**, 103.

HEIDELBERGER, C. (1964) Studies on the Molecular Mechanisms of Hydrocarbon Carcinogenesis. *J. cell. Comp. Physiol.*, **64**, Suppl. 1, 129.

HEIDELBERGER, C. (1970) Studies on the Cellular and Molecular Mechanisms of Hydrocarbon Carcinogenesis. *Eur. J. Cancer*, **6**, 161.

HEIDELBERGER, C. & IYPE, P. T. (1967) Malignant Transformation *in vitro* by Carcinogenic Hydrocarbons. *Science, N.Y.*, **155**, 214.

HERROLD, K. M. (1966) *In vivo* Carcinogenicity of Methyl Nitrosourea. *J. Path. Bact.*, **92**, 35.

KAO, F. T. & PUCK, T. T. (1971) Genetics of Somatic Mammalian Cells XII: Metagenesis by Carcinogenic Nitroso Compounds. *J. cell. Physiol.*, **78**, 139.

KIT, S., DUBBS, D. R., PIEKARSKI, L. J. & HSU, T. C. (1963) Deletion of Thymidine Kinase Activity from L Cells Resistant to Bromodeoxyuridine. *Expl Cell Res.*, **31**, 297.

KLEIHUES, P. & MAGEE, P. N. (1973) Inhibition of Protein Synthesis by N-methyl-N-nitrosourea *in vivo*. *Biochem. J.*, **136**, 303.

LAWLEY, P. D. (1966) Effects of some Chemical Mutagens and Carcinogens on Nucleic Acids. In *Prog. Nucl. Acid Res. Molec. Biol.*, **5**, 89.

LEAVER, D. D., SWANN, P. F. & MAGEE, P. N. (1969) The Induction of Tumours in the Rat by a Single Oral Dose of N-nitrosomethylurea. *Br. J. Cancer*, **23**, 177.

LITTLEFIELD, J. W. (1965) Studies on Thymidine Kinase in Cultural Mouse Fibroblasts. *Biochim. biophys. Acta*, **95**, 14.

LOVELESS, A. (1969) Possible Relevance of 0–6 Alkylation of Deoxyguanosine to the Mutagenicity and Carcinogenicity of Nitrosamines and Nitrosoamides. *Nature, Lond.*, **223**, 206.

LOVELESS, A. & HAMPTON, C. L. (1969) Inactivation and Mutation of Coliphage T2 by N-methyl- and N-ethyl-nitrosourea. *Mutation Res.*, **7**, 1.

MAGEE, P. N. & BARNES, J. M. (1967) Carcinogenic Nitroso Compounds. *Adv. Cancer Res.*, **10**, 164.

MEYER, H. M. JR, HOPPS, H. E., ROGERS, N. G., BROOKS, B. E., BERNHEIM, B. C., JONES, W. P., NISALAK, A. & DOUGLAS, R. D. (1962) Establishment of a Tissue Culture Cell Line from an African Green Monkey Kidney. *J. Immun.*, **88**, 796.

NAHA, P. M. (1969) Temperature Sensitive Conditional Mutants of Monkey Kidney Cells. *Nature, Lond.*, **223**, 1380.

NAHA, P. M. (1973*a*) Controlled Expression of the SV40 Genome. *Nature, New Biol.*, **245**, 266.

NAHA, P. M. (1973*b*) Temperature Sensitive Cells in the Study of SV40 Lysis *versus* SV40 Transformation. *Expl Cell Res.*, **80**, 467.

NAHA, P. M. (1973*c*) Early Functional Mutants of Mammalian Cells. *Nature, New Biol.*, **241**, 13.

ROSENKRANZ, H. S., BITOON, M. & SCHMIDT, R. M. (1968) Biological and Metabolic Effects of Nitrosomethylurea and Nitrosomethylurethane. *J. natn. Cancer Inst.*, **41**, 1099.

SANDERS, F. K. & BURFORD, B. O. (1967) Morphological Conversion of Cells *in vitro* by N-Nitrosomethylurea. *Nature, Lond.*, **213**, 1171.

SCHOENTAL, R. (1967) Carcinogenic Action of Nitroso Compounds. *Biochem. J.*, **102**, 5c.

SWANN, P. F. & MAGEE, P. N. (1968) Nitrosamine-induced Carcinogenesis. *Biochem. J.*, **110**, 39.


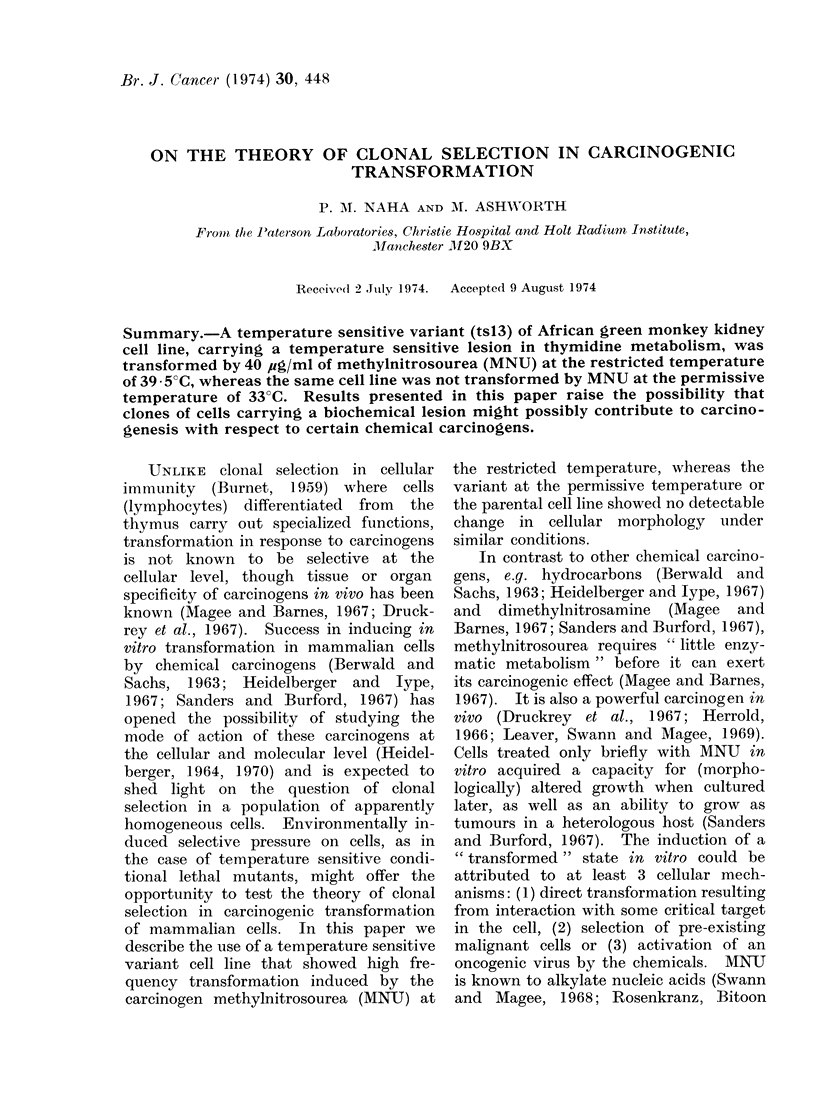

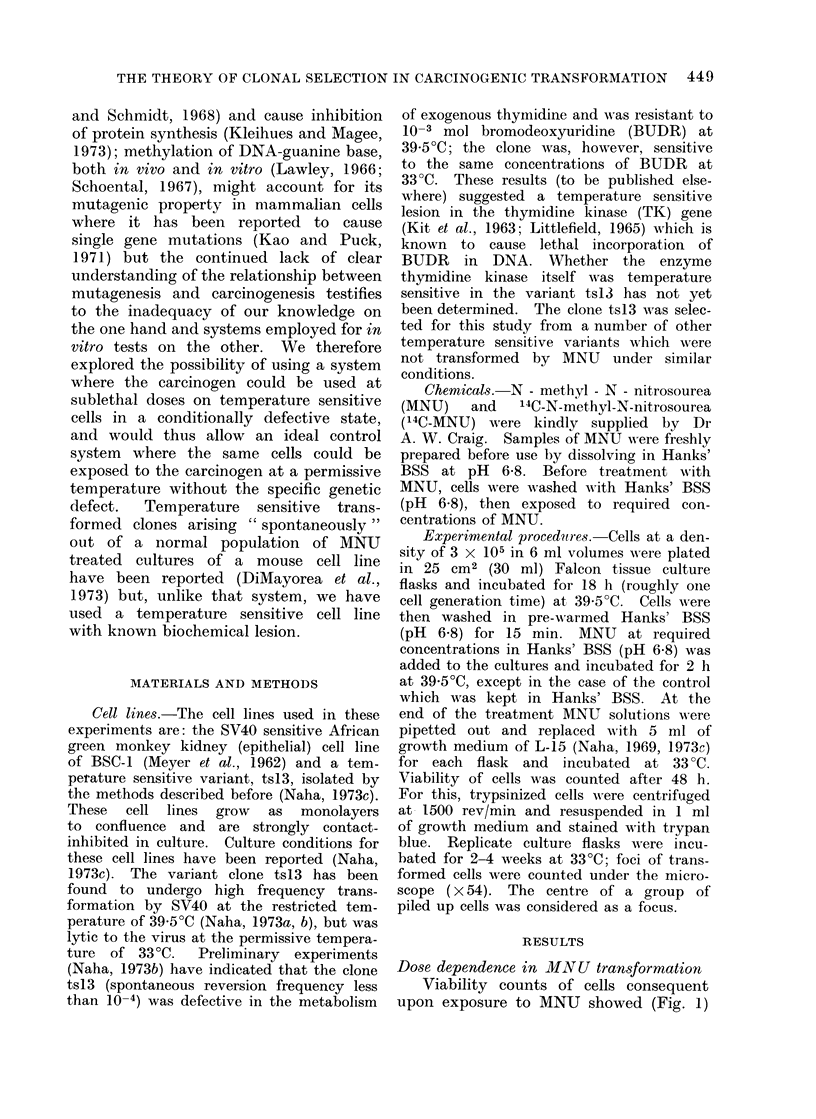

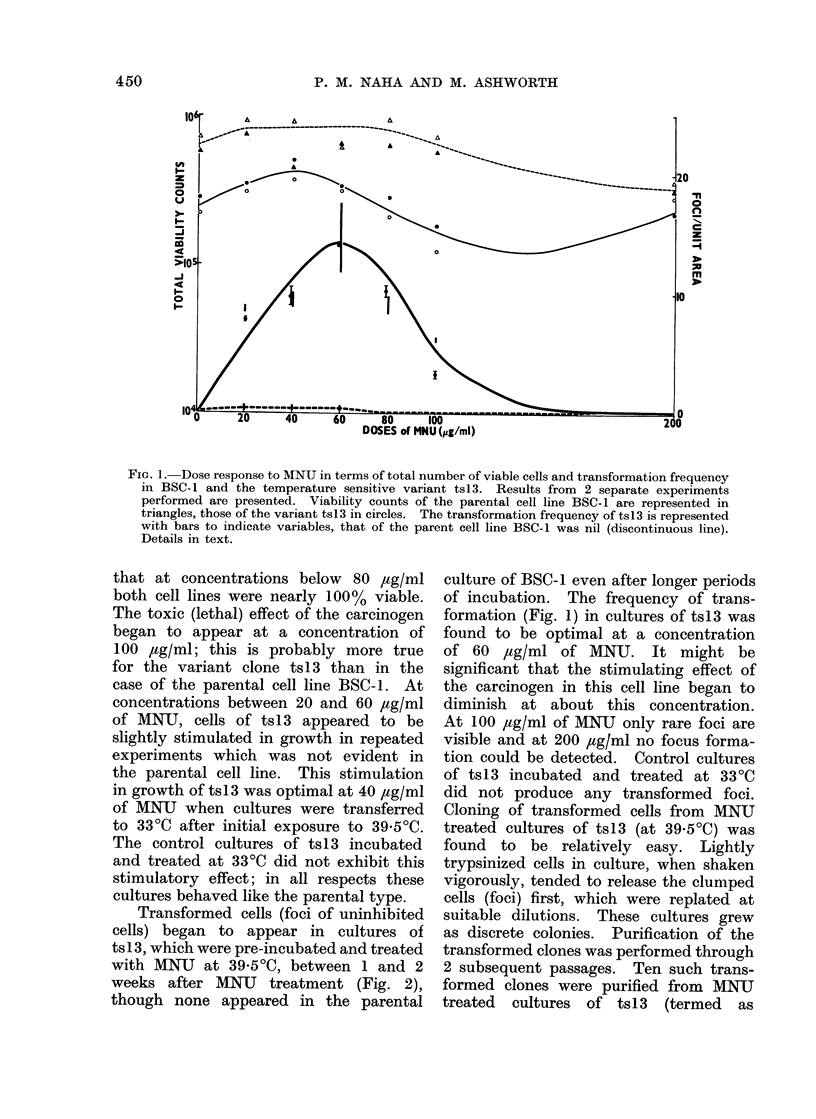

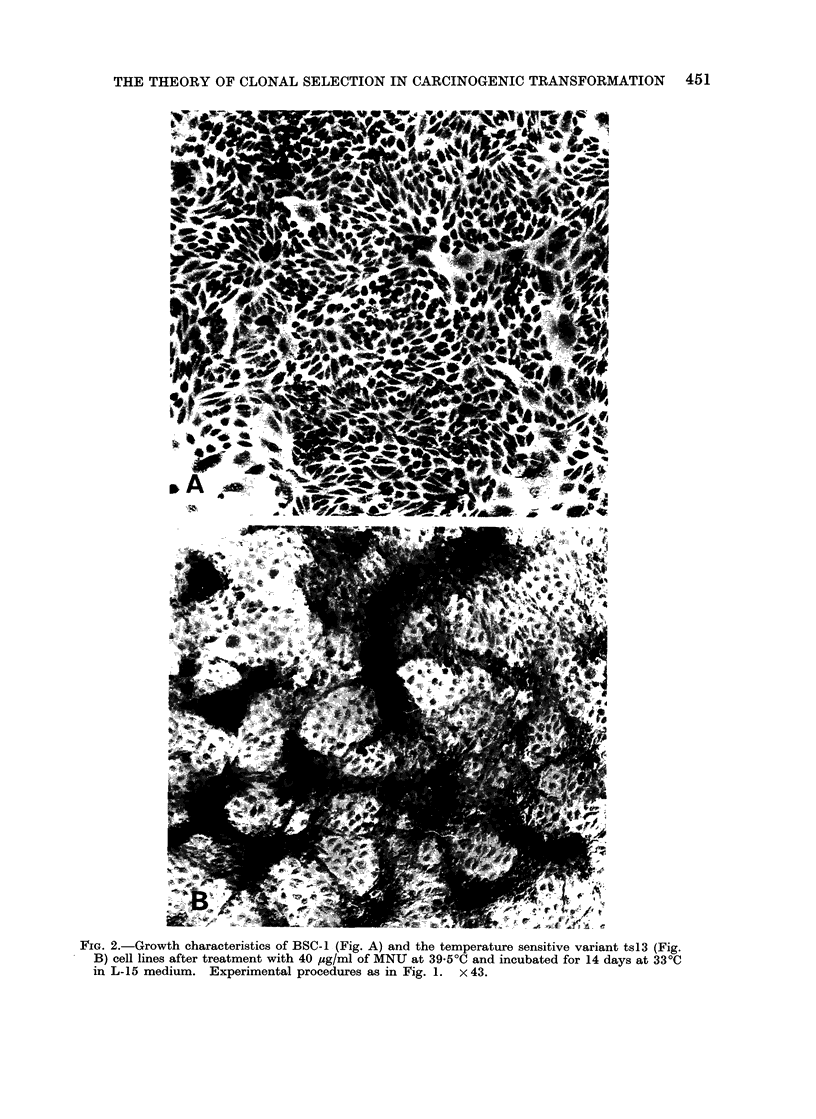

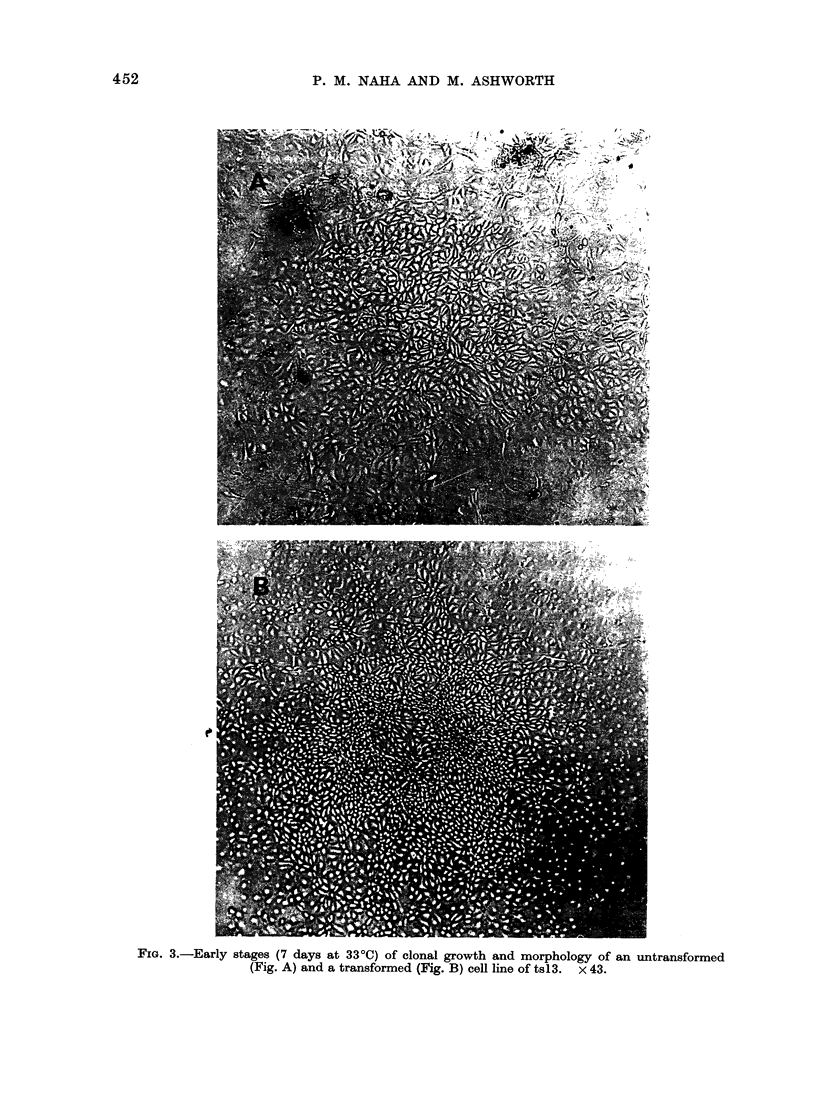

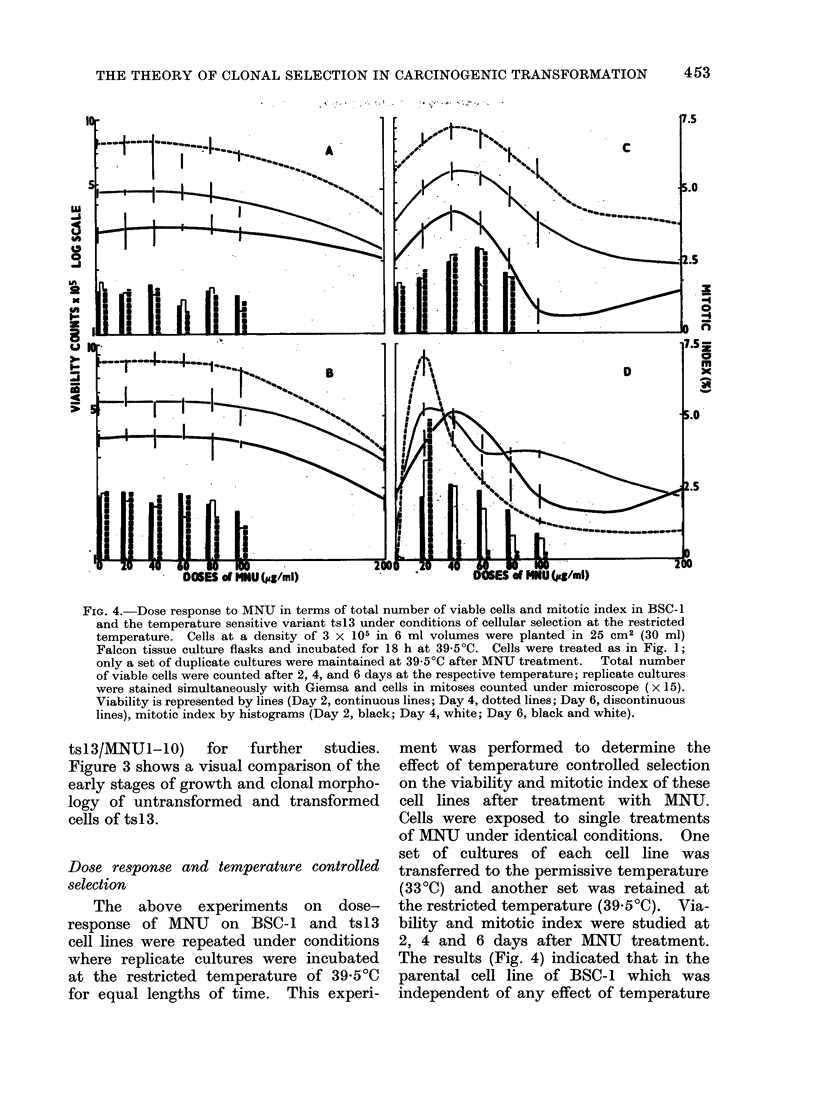

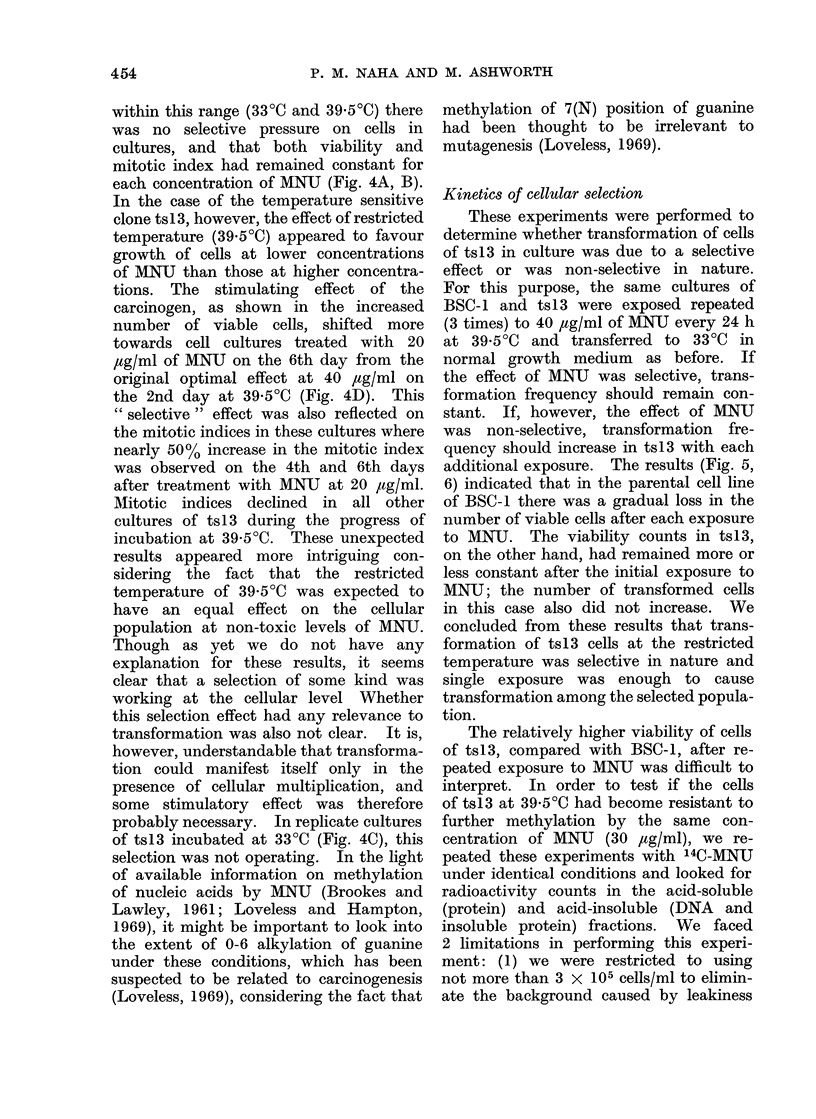

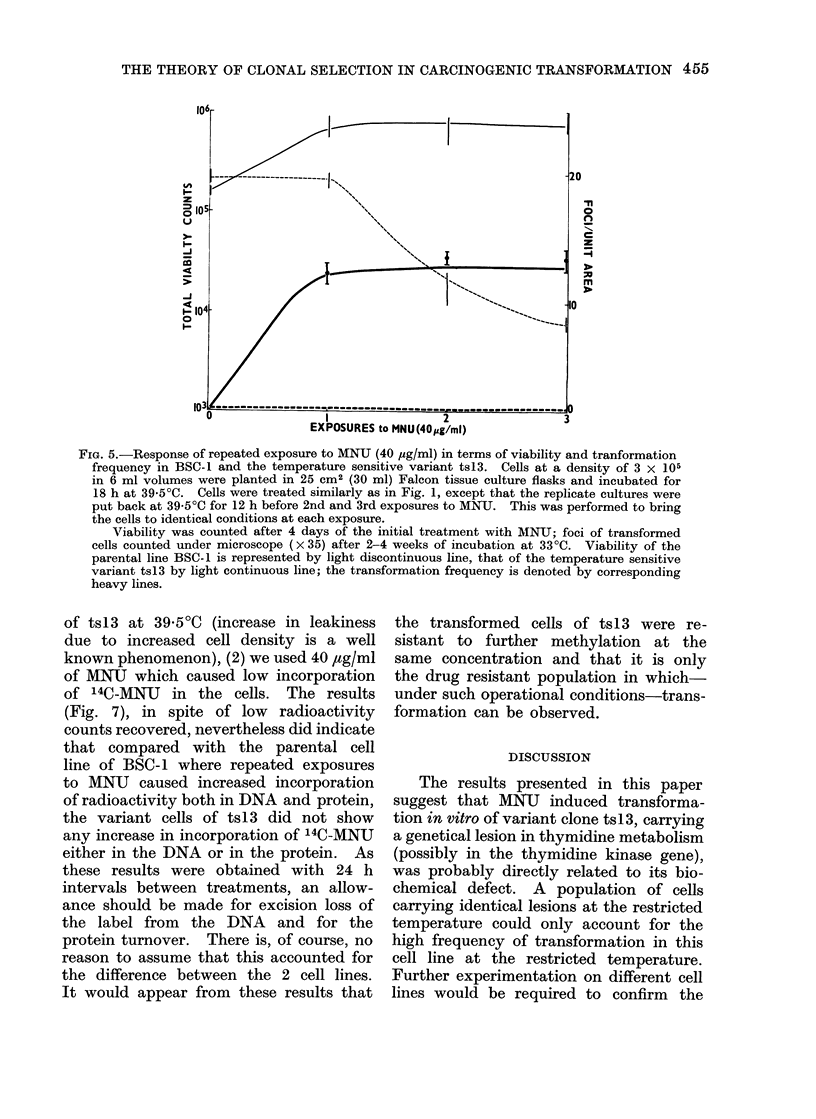

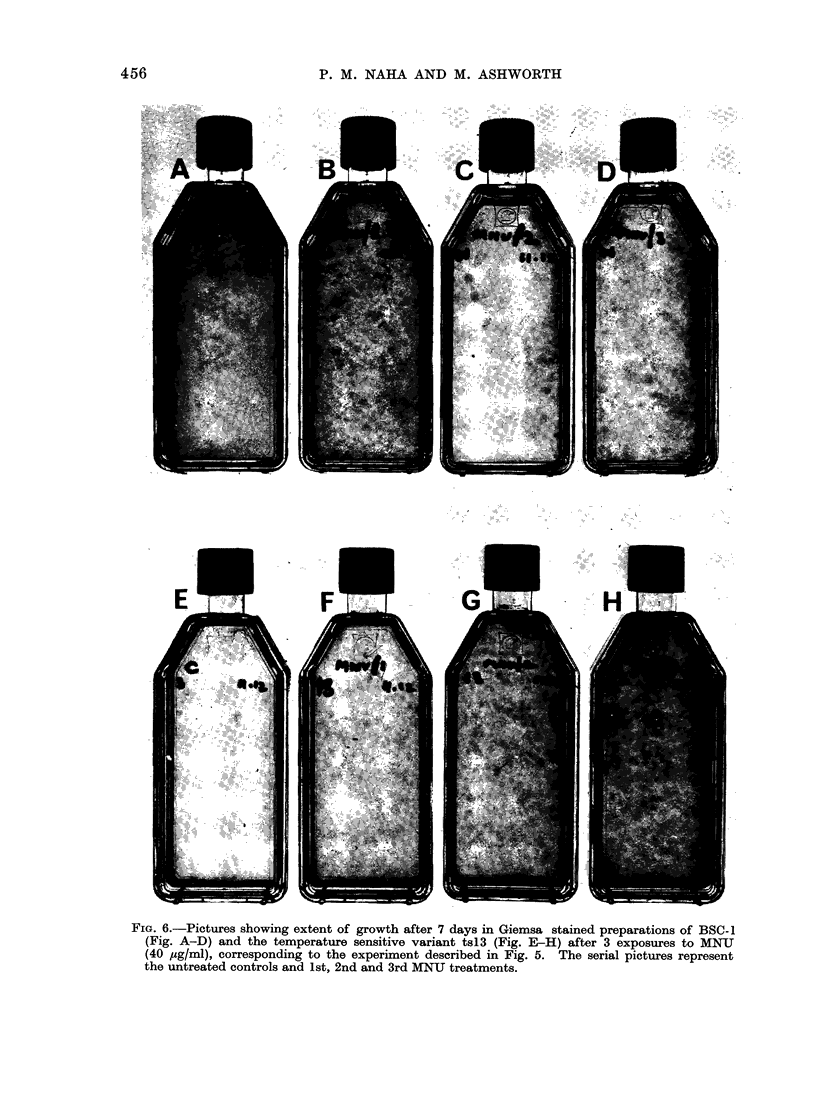

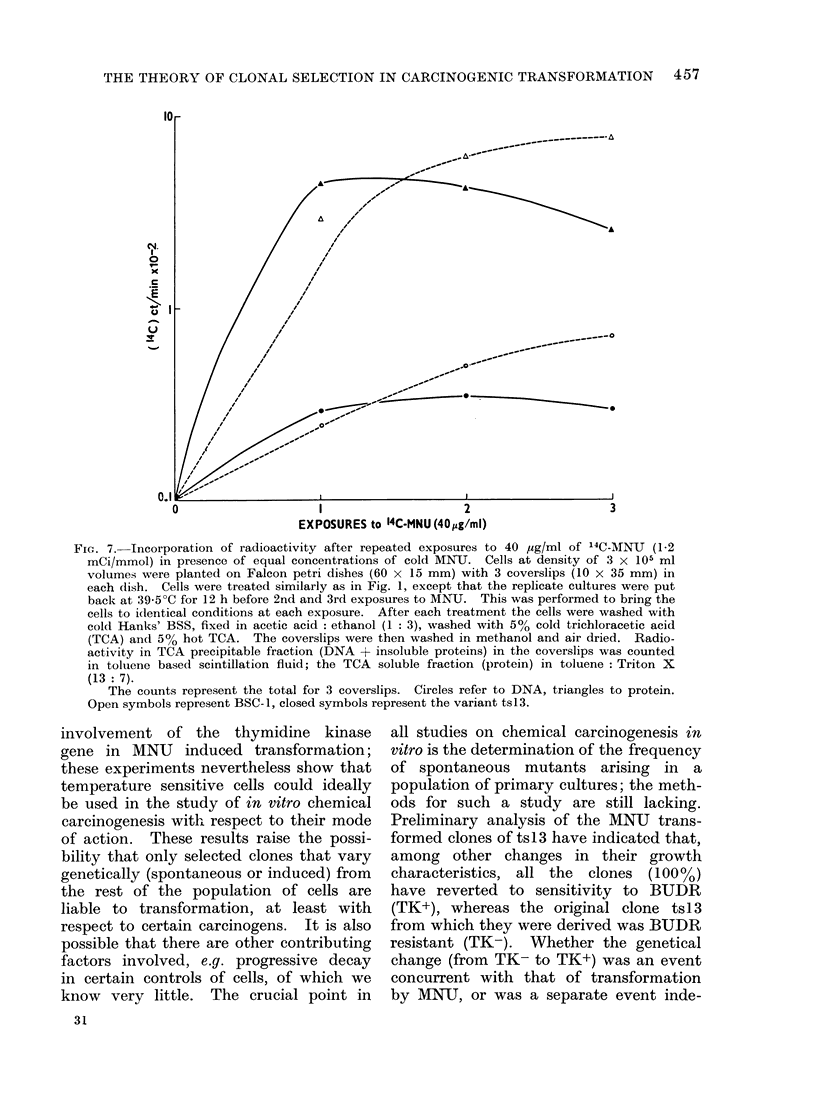

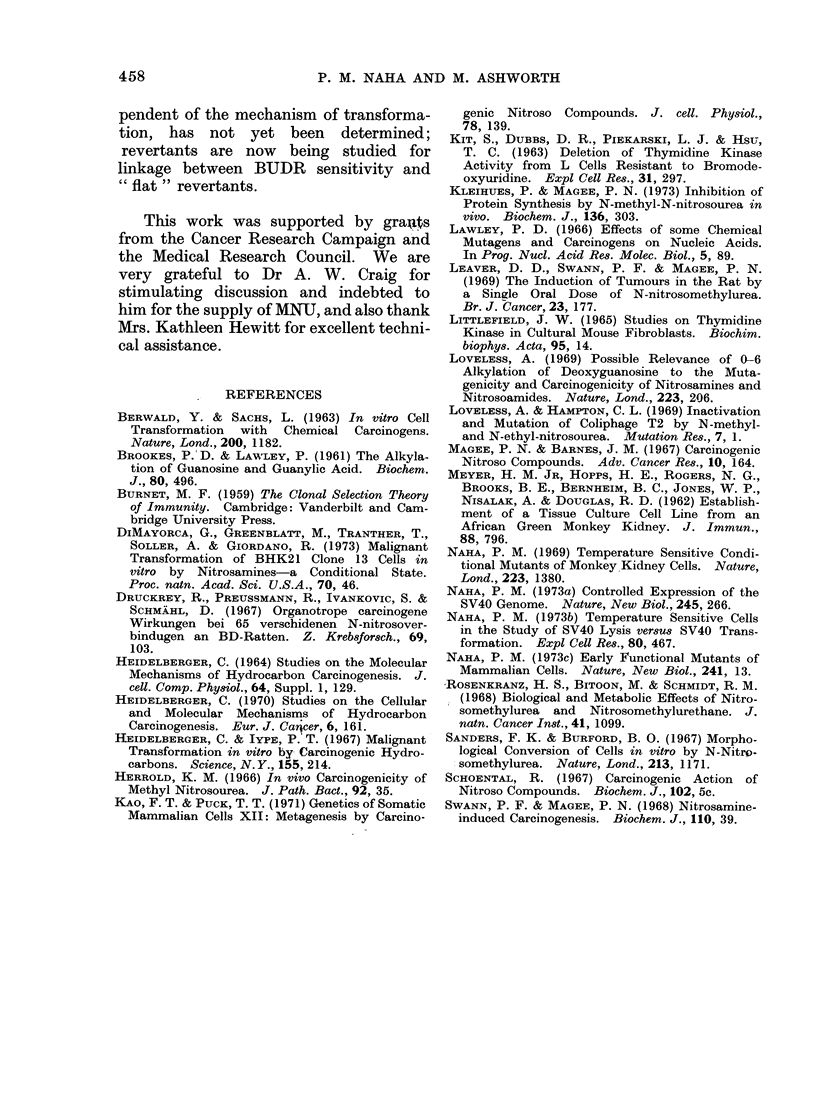


## References

[OCR_00664] BERWALD Y., SACHS L. (1963). IN VITRO CELL TRANSFORMATION WITH CHEMICAL CARCINOGENS.. Nature.

[OCR_00679] Di Mayorca G., Greenblatt M., Trauthen T., Soller A., Giordano R. (1973). Malignant transformation of BHK21 clone 13 cells in vitro by nitrosamines--a conditional state.. Proc Natl Acad Sci U S A.

[OCR_00686] Druckrey H., Preussmann R., Ivankovic S., Schmähl D. (1967). Organotrope carcinogene Wirkungen bei 65 verschiedenen N-Nitroso-Verbindungen an BD-Ratten.. Z Krebsforsch.

[OCR_00703] Heidelberger C., Iype P. T. (1967). Malignant transformation in vitro by carcinogenic hydrocarbons.. Science.

[OCR_00698] Heidelberger C. (1970). Studies on the cellular and molecular mechanisms of hydrocarbon carcinogenesis.. Eur J Cancer.

[OCR_00708] Herrold K. M. (1966). Carcinogenic effect of N-methyl-N-nitrosourea administered subcutaneously to Syrian hamsters.. J Pathol Bacteriol.

[OCR_00719] KIT S., DUBBS D. R., PIEKARSKI L. J., HSU T. C. (1963). DELETION OF THYMIDINE KINASE ACTIVITY FROM L CELLS RESISTANT TO BROMODEOXYURIDINE.. Exp Cell Res.

[OCR_00712] Kao F. T., Puck T. T. (1971). Genetics of somatic mammalian cells. XII. Mutagenesis by carcinogenic nitroso compounds.. J Cell Physiol.

[OCR_00725] Kleihues P., Magee P. N. (1973). Inhibition of protein synthesis by N-methyl-N-nitrosourea in vivo.. Biochem J.

[OCR_00741] LITTLEFIELD J. W. (1965). STUDIES ON THYMIDINE KINASE IN CULTURED MOUSE FIBROBLASTS.. Biochim Biophys Acta.

[OCR_00730] Lawley P. D. (1966). Effects of some chemical mutagens and carcinogens on nucleic acids.. Prog Nucleic Acid Res Mol Biol.

[OCR_00735] Leaver D. D., Swann P. F., Magee P. N. (1969). The induction of tumours in the rat by a single oral dose of N-nitrosomethylurea.. Br J Cancer.

[OCR_00752] Loveless A., Hampton C. L. (1969). Inactivation and mutation of coliphage T2 by N-methyl-and N-ethyl-N-nitrosourea.. Mutat Res.

[OCR_00746] Loveless A. (1969). Possible relevance of O-6 alkylation of deoxyguanosine to the mutagenicity and carcinogenicity of nitrosamines and nitrosamides.. Nature.

[OCR_00761] MEYER H. M., HOPPS H. E., ROGERS N. G., BROOKS B. E., BERNHEIM B. C., JONES W. P., NISALAK A., DOUGLAS R. D. (1962). Studies on simian virus 40.. J Immunol.

[OCR_00774] Naha P. M. (1973). Controlled expression of SV40 genome.. Nat New Biol.

[OCR_00783] Naha P. M. (1973). Early functional mutants of mammalian cells.. Nat New Biol.

[OCR_00778] Naha P. M. (1973). Temperature sensitive cells in the study of SV40 lysis versus SV40 transformation.. Exp Cell Res.

[OCR_00769] Naha P. M. (1969). Temperature sensitive conditional mutants of monkey kidney cells.. Nature.

[OCR_00787] Rosenkranz H. S., Bitoon M., Schmidt R. M. (1968). Biological and metabolic effects of nitro-somethylurea and nitrosomethylurethan.. J Natl Cancer Inst.

